# A new species of *Athelges* (Crustacea, Isopoda, Epicaridea, Bopyridae) from Japan, with morphological and DNA barcode data

**DOI:** 10.3897/zookeys.1270.160197

**Published:** 2026-02-25

**Authors:** Haruki Shinoda, Hiroki Fujita, Kenji Toyota, Tomoyuki Nakano, Michitaka Shimomura

**Affiliations:** 1 Graduate School of Science, Kyoto University, Kitashirakawa-Oiwake, Kyoto, 606-8502, Japan Kanagawa University Yokohama Japan; 2 Seto Marine Biological Laboratory, Field Science Education and Research Center, Kyoto University, 459 Shirahama, Wakayama, 649-2211, Japan Graduate School of Science, Kyoto University Kyoto Japan; 3 Department of Bioresource Science, Graduate School of Integrated Sciences for Life, Hiroshima University, 1-4-4 Kagamiyama, Higashihiroshima, Hiroshima, 739-8528, Japan Hiroshima University Higashihiroshima Japan; 4 Department of Biological Sciences, Faculty of Science, Kanagawa University, 3-27-1, Rokkakubashi, Kanagawa, Yokohama, Kanagawa, 221-8686, Japan Tokyo University of Science Tokyo Japan; 5 Department of Biological Science and Technology, Faculty of Industrial Science and Technology, Tokyo University of Science, 6-3-1 Niijuku, Katsushika, Tokyo, 125-8585, Japan Seto Marine Biological Laboratory, Field Science Education and Research Center, Kyoto University Shirahama Japan

**Keywords:** Asia, Athelginae, Diogenidae, Isopoda, Paguroidea, parasite

## Abstract

*Athelges pileus***sp. nov**. is described as an abdominal bopyrid of the hermit crab *Diogenes edwardsii* collected from the Noto Peninsula, Japan. *Athelges pileus***sp. nov**. is distinguished from its congeners by the following combination of characters. Female: head deeply sunk in thorax, anterolateral margin of head with pair of notches; anterior margin of frontal lamina concave; merus of pereopod 1 with broad and long projection ventrally; pereopods 5 and 6 with large gap between them; pleomeres 1–4 each with subovate biramous pleopods; each pleopod with peduncle, becoming smaller posteriorly; pleotelson with mushroom-shaped end. Male: pleon subovate with anal cone distally. The present species is the 14^th^ member of the genus *Athelges*, and the second species from Asia. This is the second species of *Athelges* infesting *Diogenes edwardsii*. We also provide a partial sequence of the mitochondrial cytochrome c oxidase subunit 1 (COI) gene (658 bp), mitochondrial 16S rRNA gene (482 bp), and nuclear 18S rRNA (724 bp) as a DNA barcode for the species. We show by molecular phylogenetic analysis that the present species is distinct from *A. takanoshimensis*.

## Introduction

Athelginae Codreanu & Codreanu, 1956 is one of the subfamilies in Bopyridae Rafinesque, 1815 and is composed of abdominal ectoparasites of species in Paguroidea; 45 species in nine genera have been reported worldwide (Boyko et al. 2025a; Shinoda et al. 2025). *Athelges* Gerstaecker, 1862 exhibits the highest species diversity within Athelginae, comprising 13 species (Boyko et al. 2025b): *Athelges aegyptius* Codreanu, Codreanu & Pike, 1965; *A. ankistron* Markham, 2010; *A. bilobus* G.O. Sars, 1898; *A. caudalis* Barnard, 1955; *A. cladophorus* Gerstaecker, 1862; *A. guitarra* Giard & Bonnier, 1890; *A. intermedia* (Anonymous, 1886); *A. lacertosi* Pike, 1961; *A. paguri* (Rathke, 1843); *A. pelagosae* Babić, 1912; *A. prideauxii* Giard & Bonnier, 1890; *A. takanoshimensis* Ishii, 1914; *A. tenuicaudis* G.O. Sars, 1898.

Species belonging to the genus have been recorded from the northeastern Atlantic, the western Indian Ocean, and the western Pacific. (Golovan and Malyutina 2010; An et al. 2022). The genus *Athelges* has been reported with eight species (*A. bilobus*, *A. cladophorus*, *A. guitarra*, *A. intermedia*, *A. paguri*, *A. pelagosae*, *A. prideauxii*, *A. tenuicaudis*) from Europe (Adriatic Sea; Britain; Copenhagen; Denmark; France; Italy; North Atlantic; Norway; Spain), two (*A. aegyptius*, *A. caudalis*) from Africa (Egypt; Mozambique), and two (*A. ankistron*, *A. lacertosi*) from Oceania (Australia; New Zealand). Among the species of *Athelges*, only *Athelges takanoshimensis* Ishii, 1914 has been recorded from Asian localities, including: Russia (Vostok Bay and Peter the Great Gulf), Korea, China, Taiwan, Japan, Philippines, Singapore, Indonesia, and Papua New Guinea (Nagasawa 2020; An et al. 2022).

The number of hermit crab species reported as hosts of the genus *Athelges* reaches 36, across 10 genera in two families, Diogenidae and Paguridae (An et al. 2022). Four species are known from *Anapagurus*, one from *Calcinus*, one from *Ciliopagurus*, one from *Clibanarius*, one from *Dardanus*, four from *Diogenes*, four from *Lophopagurus*, three from *Paguristes*, one from *Pagurodofleinia*, and 16 from *Pagurus*. Species of *Pagurus* are the most frequently used hosts of *Athelges*. In this study, an undescribed species of *Athelges* was collected from Japan.

This species was found on the dorsal surface of the pleon of a hermit crab identified as *Diogenes edwardsii* (De Haan, 1849) caught in a basket trap for whelk fishing in the offshore waters of Sosogi, Wajima, Ishikawa Prefecture in 2023. Based on this material, we describe a new species of *Athelges* and provide the mitochondrial cytochrome c oxidase subunit I (COI) gene and 16S rRNA gene, as well as the nuclear 18S rRNA gene barcode sequences to aid future DNA barcoding in species of *Athelges*. We showed that the present species is distinct from *A. takanoshimensis* based on molecular phylogenetic analysis The present species is the 14^th^ member of the genus, and the second species recorded from Asia. This is a second species of *Athelges* infesting *Diogenes edwardsii*.

## Materials and methods

### Sample collection and morphological observation

A total of 349 specimens of the hermit crab *Diogenes edwardsii* were collected by a basket trap for whelk fishing at a depth of approximately 13 m off the Noto Peninsula, Japan in 2023. After hermit crabs were removed from gastropod shells by cracking them with a hammer, the pleons were examined to check for the presence of parasitizing bopyrids. Two hermit crabs were infested on the pleon by female bopyrid isopods, one of which was accompanied by a male. A total of three samples retained were fixed and preserved in 100% ethanol. The bopyrids were removed from the host under a stereomicroscope SMZ 1270 (Nikon, Tokyo, Japan). Each individual was dissected and prepared for morphological and anatomical observation using a stereomicroscope SMZ 1270 (Nikon, Tokyo, Japan) and biological microscope ECLIPSE E600 (Nikon) with drawing tubes. Total length (**TL**) was measured from the anterior margin of the head to the posterior margin of the pleotelson. Shield length (**SL**) is provided as an indicator of size for the host crab. Terminology follows Williams and Boyko (2016). The type series is deposited in the Seto Marine Biological Laboratory (**SMBL**).

### DNA extraction, PCR and sequencing

We performed molecular analyses on two females, three specimens of *Athelges takanoshimensis* and two species of Pseudioninae for use as outgroups. Details of the species used in this study are listed in Table 2. Total DNA was extracted using a High Pure PCR Template Preparation Kit (Roche, Basel, Switzerland). Total DNA was used as a template for amplifying three molecular markers: the mitochondrial COI and 16S rRNA genes, as well as the nuclear 18S rRNA gene. Primers for the polymerase chain reaction (PCR) are listed in Table 1. PCR amplification was performed using Ex Taq (Takara, Shiga, Japan) in a 25 µL reaction volume. The cycling conditions, adapted from Karasawa et al. (2014), consisted of an initial denaturation at 94 °C for 3 min, followed by 30 cycles of denaturation at 94 °C for 1 min, annealing at 46 °C for 1 min, and extension at 72 °C for 1 min, with a final extension at 72 °C for 7 min. The PCR products were purified using the ExoSAP-IT PCR Product Cleanup Reagent (Thermo Fisher Scientific, Waltham, MA, USA). Purified PCR products were submitted to the DNA sequencing service of Eurofins Genomics Inc (Tokyo, Japan). Purified PCR products were sequenced in both directions. Sequences were assembled using ATGC v. 7.02 (Nihon Server, Tokyo, Japan).

**Table 1.** Primers used for PCR amplification and sequencing of the mitochondrial COI, 16S rRNA genes and nuclear 18S rRNA genes.

Primer	Direction	Sequence 5’–3’	Reference
COI			
PCR amplification and sequencing			
LCO1490	Forward	GGTCAACAAATCATAAAGATATTGG	Folmer et al. (1994)
HCO2198	Reverse	TAAACTTCAGGGTGACCAAAAAATCA	Folmer et al. (1994)
16S rRNA			
PCR amplification and sequencing			
ISO16SF	Forward	TCGCCTGTTTAACAAAAACA	Fukuchi, unpublished
ISO16SR	Reverse	CGGTCTGAACTCAAATCATG	Fukuchi, unpublished
18S rRNA			
PCR amplification and sequencing			
18Sforward	Forward	TACCTGGTTGATCCTGCCAG	Maraun et al. (2009)
18S614r	Reverse	TCCAACTACGAGCTTTTTAACC	Maraun et al. (2009)

**Table 2.** List of analyzed specimens with their specimen number, type status, host, collection locality, date, sex, and GenBank accession number.

Species	Specimen no.	Type status	Host species	Sampling locality in Japan	Coll. Date	Sex	Accession no.
*Asymmetrione asymmetrica* (Shiino, 1933)	SMBL-V0863		*Clibanarius virescens* (Krauss, 1843)	Wakayama, Japan	15 Nov. 2023	Female	LC903005, LC903010, LC903015
*Athelges pileus* sp. nov.	SMBL-V0856	Holotype	*Diogenes edwardsii* (De Haan, 1849)	Ishikawa, Japan	31 Jul. 2023	Female	LC876712.1, LC876714.1, LC876786.1
	SMBL-V0857	Allotype	*Diogenes edwardsii* (De Haan, 1849)	Ishikawa, Japan	31 Jul. 2023	Male	–
	SMBL-V0858	Paratype	*Diogenes edwardsii* (De Haan, 1849)	Ishikawa, Japan	31 Jul. 2023	Female	LC876713.1, LC876715.1, LC876787.1
*Athelges takanoshimensis* Ishii, 1914	SMBL-V0864		*Pagurus filholi* (De Man, 1887)	Nagasaki, Japan	27 Sep. 2023	Female	LC903007, LC903012, LC903017
	SMBL-V0865		*Pagurus filholi* (De Man, 1887)	Nagasaki, Japan	27 Sep. 2023	Female	LC903008, LC903013, LC903018
	SMBL-V0866		*Pagurus filholi* (De Man, 1887)	Nagasaki, Japan	27 Sep. 2023	Female	LC903009, LC903014, LC903019
*Eremitione clibanaricola* (Shiino, 1933)	SMBL-V0867		*Clibanarius virescens* (Krauss, 1843)	Wakayama, Japan	15 Nov. 2023	Female	LC903006, LC903011, LC903016

### Phylogenetic analysis

Phylogenetic analyses were performed using sequences of three genes obtained from eight individuals. Each gene was aligned with MAFFT v. 7 on the web server (Katoh and Standley 2013; Katoh et al. 2019). For the 16S and 18S rRNA datasets, gaps and ambiguously aligned sites were removed using Gblocks v. 0.91b (Castresana 2000). The final alignment lengths were 451 bp for 16S rRNA and 642 bp for 18S rRNA. The three gene datasets were concatenated, resulting in a total length of 1751 bp. Maximum likelihood (ML) phylogenetic trees were constructed using IQ-TREE web server (Nguyen et al. 2015; Trifinopoulos et al. 2016). The optimal substitution model was selected automatically by IQ-TREE. Nodal support was estimated using 1,000 ultrafast bootstrap replicates (UFBoot) and 1,000 SH-like approximate likelihood ratio tests (SH-aLRT). Nodes with SH-aLRT ≥ 80% and UFBoot ≥ 95% were considered well supported. The resulting phylogenetic tree was drawn with FigTree software v. 1.4.4. Kimura (1980) 2-parameter (K2P) distances for the COI gene among the five *Athelges* sequences were calculated using MEGA7 (Kumar et al. 2016).

## Results

### Family Bopyridae

**Subfamily Athelginae Codreanu & Codreanu, 1956**

**Genus *Athelges* Gerstaecker, 1862**

#### Athelges pileus

Taxon classification>AnimaliaIsopodaBopyridae

Shinoda & Shimomura sp. nov.

9663C94B-60EB-54A7-9680-15F57C01ED9A

https://zoobank.org/AC563BCC-3D6F-471A-910C-15A672CB7821

**Figure 1.**
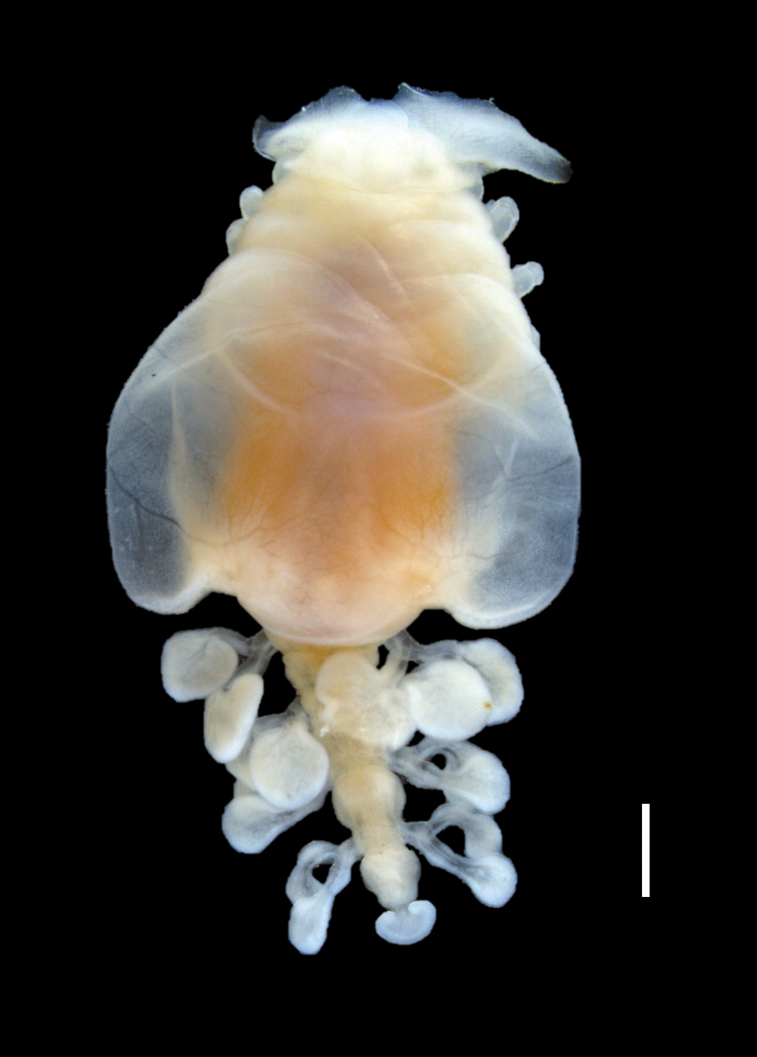
*Athelges pileus* sp. nov. holotype female, photographed. Habitus, ventral view. Scale bar: 1 mm.

**Figure 2.**
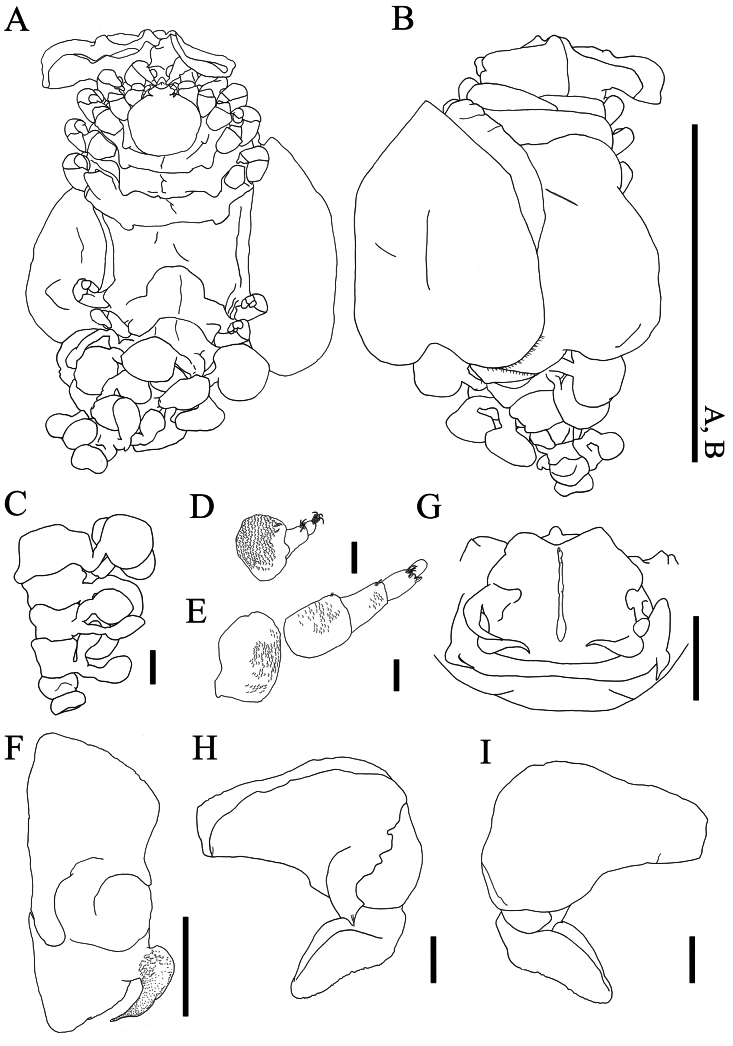
*Athelges pileus* sp. nov. holotype female. A. Habitus, dorsal view; B. Habitus, ventral view; C. Pleon, dorsal view; D. Right antennule, dorsal view; E. Right antenna, dorsal view; F. Left maxilliped, outer view; G. Head, barbula and posterior digitate extension, ventral view; H. Left oostegite 1, inner view; I. Left oostegite 1, outer view. Scale bars: 1 cm (A, B); 1 mm (C, E–H); 100 μm (D, E).

**Figure 3.**
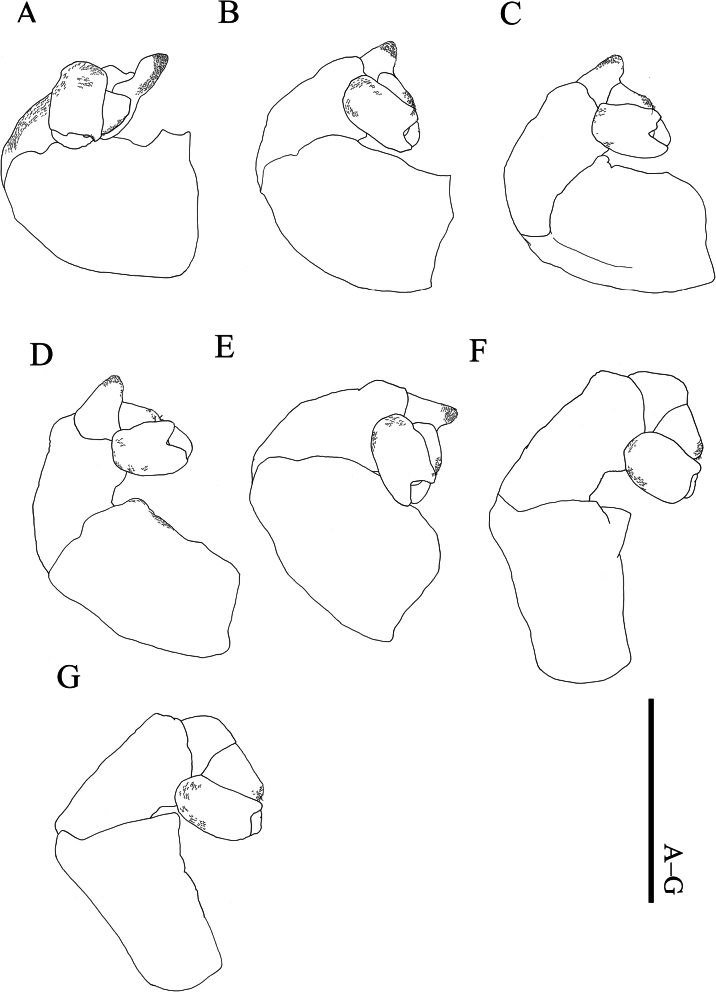
*Athelges pileus* sp. nov. holotype female. A–G. Right pereopods 1–7. Scale bar: 1 mm.

**Figure 4.**
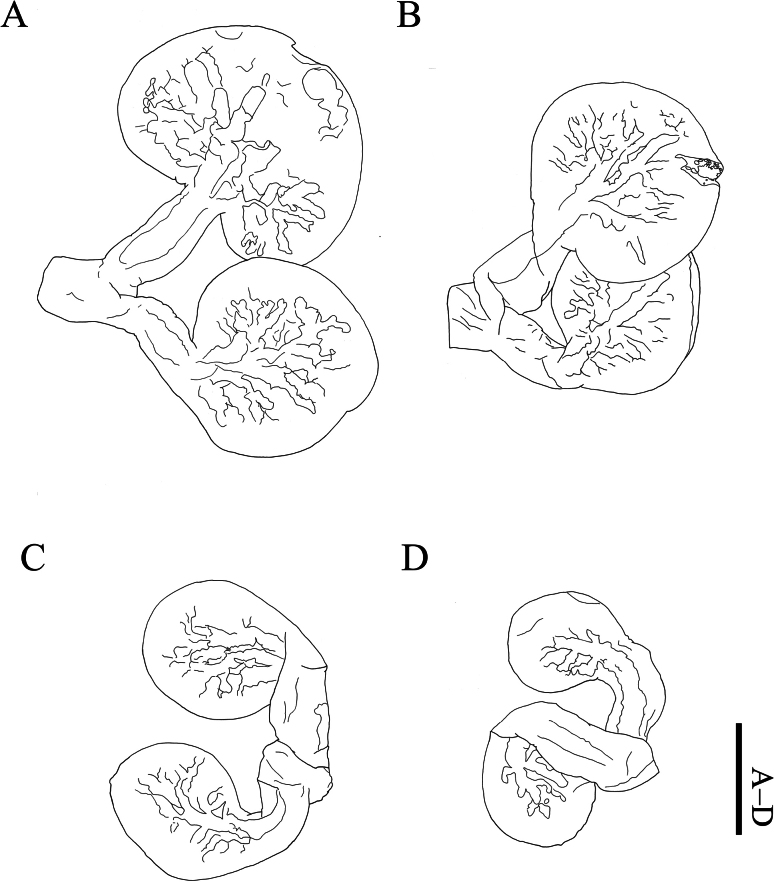
*Athelges pileus* sp. nov. holotype female. **A–D** left pleopods 1–4. Scale bar: 1 mm.

**Figure 5.**
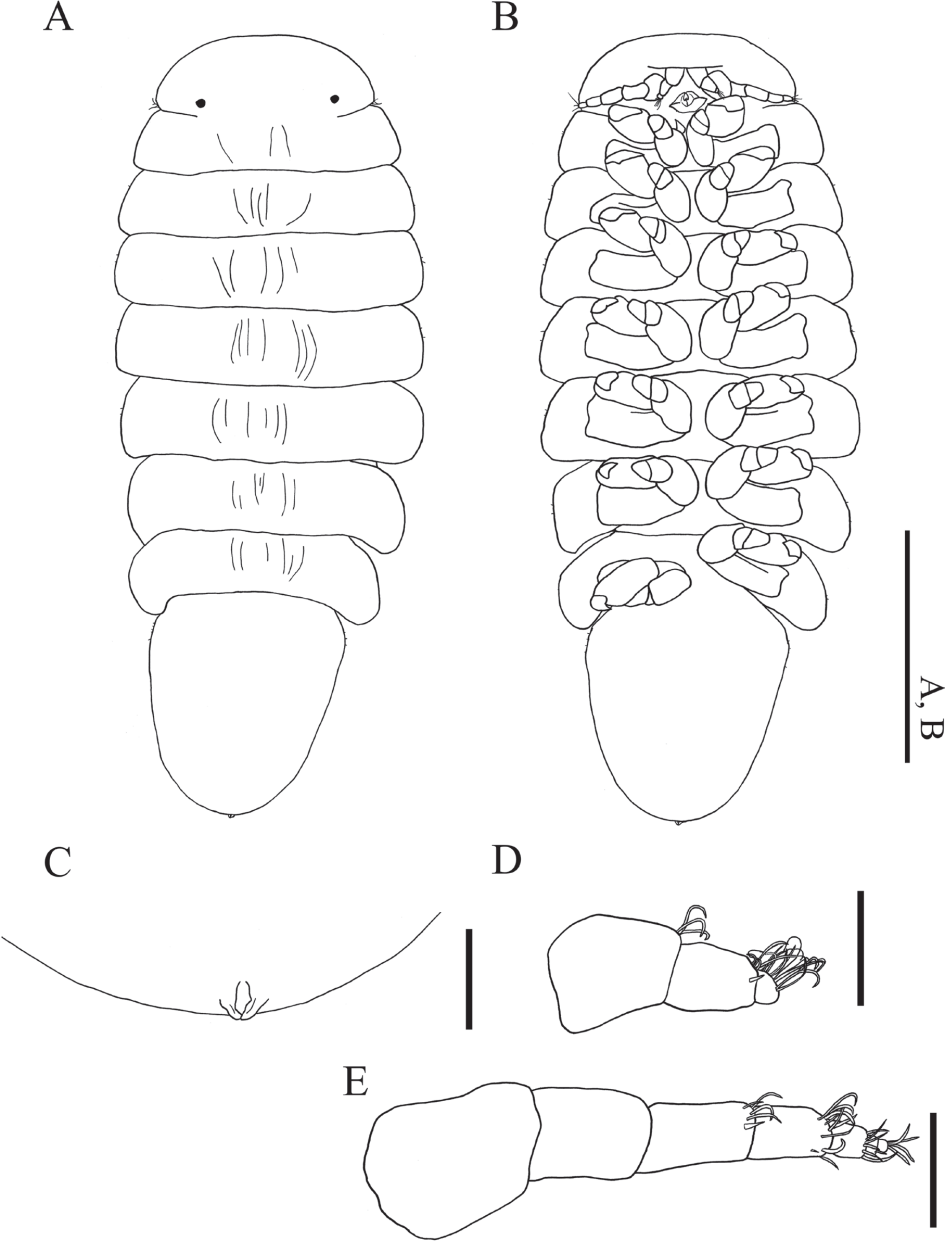
*Athelges pileus* sp. nov. allotype male. A. Habitus, dorsal view; B. Habitus, ventral view; C. Detail of posterior margin of pleon, ventral view; D. Left antennule, ventral view; E. Left antenna, ventral view. Scale bars: 1 mm (A, B); 100 μm (C–E).

**Figure 6.**
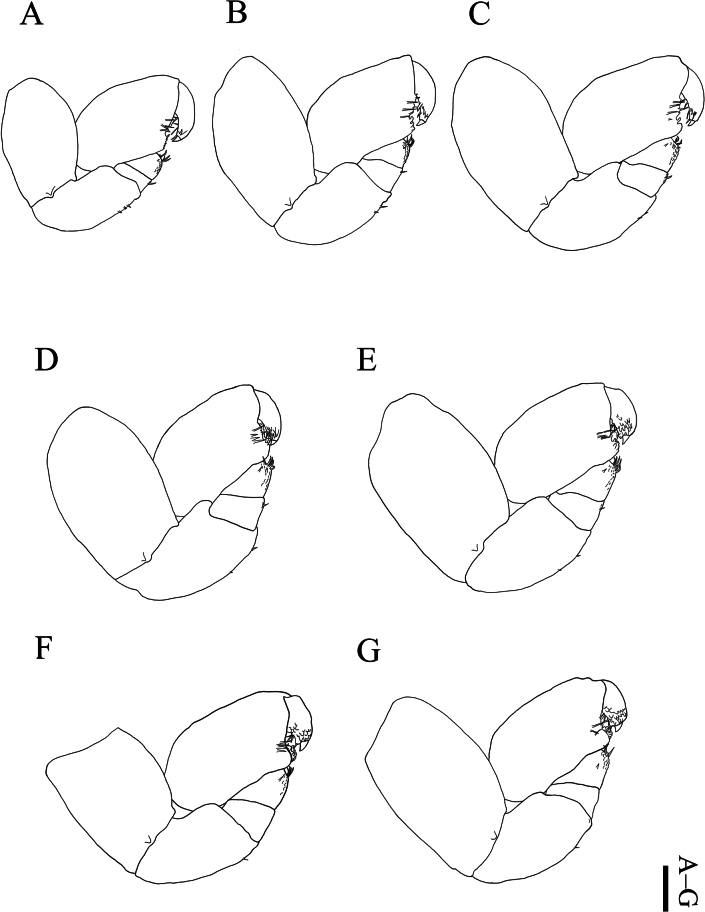
*Athelges pileus* sp. nov. allotype male. A–G. Right pereopods 1–7. Scale bar: 100 μm.

Figs 1, 2, 3, 4, 5, 6 [New Japanese name: Kinoko-o-harayadori]

##### Material examined.

***Holotype***: Japan • ♀ (TL 13.3 mm), infesting *Diogenes edwardsii* (SL 8.4 mm); Ishikawa, off the coast of Noto Peninsula, Sosogi; 37°27'36.3"N, 137°04'27.8"E; approximately 13 m depth; 31 July 2023; K. Toyota, K. Tsunoda, T. Sumi, and Tone leg. Basket trap; GenBank: accession numbers LC876712.1, LC876714.1, LC876786.1; SMBL-V0856. ***Allotype***: Japan • ♂ (TL 3.4 mm), data same as for holotype; SMBL-V0857. ***Paratype***: Japan • ♀ (TL 10.1 mm), infesting *Diogenes edwardsii* (SL 7.2 mm), data same as for holotype; GenBank: accession numbers LC876713.1, LC876715.1, LC876787.1; SMBL-V0858.

##### Diagnosis.

Female: head deeply sunk in thorax, anterior margin of frontal lamina concave; anterolateral margin of head with pair of notches; merus of pereopod 1 with broad and long projection ventrally; pereopods 5 and 6 with large gap between them; pleomeres 1–4 each with subovate biramous pleopods; each pleopod with peduncle, becoming smaller posteriorly; pleotelson with mushroom-shaped end.

Male: pleon subovate with anal cone distally.

##### Description of holotype female.

(Figs 1, 2, 3, 4) Total length 13.3 mm, maximal width 5.1 mm; head length 2.1 mm, head width 2.2 mm, pleon length 5.3 mm. Body: head and pereon nearly symmetrical, pleon slightly turning towards left. Head, pereomeres 1–7, pleomeres 1–4, pleotelson distinctly separated (Fig. 2A–C).

Head (Fig. 2A) as long as wide, with frontal lamina: posterior and lateral margins convex. Frontal lamina fused with head. Eyes lacking. Antennule (Fig. 2D) of three articles: each article with fine setae distally; article 1 with some scales. Antenna (Fig. 2E) of five articles: articles 1–3 with some scales; articles 2–4 with fine setae distally. Maxilliped (Fig. 2F) with elongate subrectangular anterior lobe lacking palp: smaller, rounded posterior lobe bearing long, thin, acute spur. Barbula (Fig. 2G) with three short, distally acute lobes laterally on each side. Posterior digitate extension (Fig. 2G) on pereomere 2.

Pereon (Fig. 2A) of seven pereomeres, broadest at pereomeres 5 and 6, tapering anteriorly and posteriorly: pereomeres 1–5 anteriorly concave and posteriorly convex; pereomeres 6 and 7 anteriorly convex and posteriorly concave. Oostegites (Fig. 2B) completely enclosing brood pouch. Oostegite 1 (Fig. 2H, I) extended over head: anterior lobe laterally expanded and folded; posterior lobe with broad anterior margin tapering to posterior rounded tip; ridge of posterior margin slightly undulate. Oostegite 5 with fringe of setae on posterior margin; each seta of oostegite 5 approximately 0.4 mm in length. All pereopods (Fig. 3A–G) with distinct segmentation: propodi each with some scales; carpi each with distoventral tuft of setae. Pereopods 1–5 (Fig. 3A–E) carpi each with bearing simple scales. Pereopod 1 (Fig. 3A) anterior to head: ischium with simple scales. Pereopods 1–3 (Fig. 3A–C) parallel to head. Pereopod 4 (Fig. 3D): ischium with simple scales. Pereopods 2–5 (Fig. 3B–E): meri each with projection distoventrally. Bases and ischia of pereopods 6 and 7 (Fig. 3F, G) longer than pereopods 1–5.

Pleon (Fig. 2A, C) of five pleomeres, pleomeres 1–4 and pleotelson dorsally distinct. Pleomere 1 with broad pleopods (Fig. 4A): endopod reniform; exopod subovate. Pleomeres 2–4 each with subovate pleopods (Fig. 4B–D). Pleotelson without uropods: distal end mushroom-shaped

##### Description of male.

(Figs 5, 6) Total length 3.4 mm, maximum width 1.3 mm; head length 0.3 mm, head width 0.9 mm, pleon length 1.0 mm. Head (Fig. 5A) subovate, widest posteriorly, fused with pereomere 1 medially. Small eyes near posterolateral margins. Antennule (Fig. 5D) of three articles: all articles with fine setae distally, article 2 with seta medially. Antenna (Fig. 5E) of seven articles: articles 3–7 with some setae distally. Pereon widest at pereomeres 4 and 5: pereomeres 1–4 slightly tapering anteriorly; pereomeres 5–7 slightly curved posterolaterally. Pereopods 2–7 (Fig. 6B–G) similar in size; pereomere 7 fused with pleon ventrally. Pereopod 1 (Fig. 6A) smaller than pereopods 2–7. All pereopods with distinct articles: dactyli each with some setae on lateral and ventral margins; propodi each with some setae and few blunt teeth on ventral margin near to insertion of tip of dactylus; carpi each with tuft of setae and some scales on distoventral margin, with seta on lateral margin. Pereopods 1–5 (Fig. 6A–E); meri each with seta on distoventral margin, ischia each with two setae on ventral margin. Pereopods 6 and 7 (Fig. 6F, G); ischia each with seta on ventral margin. Pleon (Fig. 5A, B) subovate, all pleomeres fused without lateral indication of segmentation; posterior margin rounded (Fig. 5C), anal cone distally, without pleopods.

##### Remarks.

The present species was identified as belonging to the genus *Athelges* Gerstaecker, 1862 by the lack of lateral plates and by the presence of a cylindrical pleon. *Athelges pileus* sp. nov. is similar to *Athelges ankistron* Markham, 2010 from Australia and *Athelges caudalis* Barnard, 1955 from South Africa in having the pleotelson of the female produced into a prominent, reflexed, anchor-shaped end (An et al. 2022). *Athelges pileus* sp. nov. is distinguished from *A. ankistron* by the following features (those of *A. ankistron* given in parentheses): pleon extended posteriorly in female (pleon reflexed back), anterolateral margin of head with pair of notches in female (anterolateral margin of head without notches), antenna of five articles in female (antenna of six articles), pereomeres 5 and 6 broadest in female (all pereomeres same width), carpus of pereopod 1 with broad and long projection ventrally in female (carpus of pereopod 1 with projection distoventrally), pereomeres 2–5 with anterior narrowing on dorsal surface in female (pereomeres 2–5 in nearly straight lines on dorsal surface), pereopods 5 and 6 with large gap between them in female (without large gap), pleotelson without uropods, with mushroom-shaped end in female (pleotelson produced into two reflexed posterolateral lobes), antenna of seven articles in male (antenna of five articles), and pleon anteriorly narrower than pereomere 7 in male (pleon anteriorly nearly as broad as pereomere 7). *Athelges pileus* sp. nov. is distinguished from *A. caudalis* by the following features (those of *A. caudalis* given in parentheses): pleotelson without uropod, with mushroom-shaped end in female (pleotelson produced into two terminal semicircular posterolateral lobes).

The present species is similar to *A. takanoshimensis* in having a maxilliped with elongate subrectangular anterior lobe lacking a palp and smaller, rounded posterior lobe bearing long, thin, acute spur in female, oostegite 1 extended overhead with anterior lobe laterally expanded, folded and posterior lobe with broad anterior margin tapering to posterior rounded tip in female and pleotelson with anal cone in male. *Athelges pileus* sp. nov. is distinguished from *A. takanoshimensis* by the following features (those of *A. takanoshimensis* in parentheses): pleon extended posteriorly in female (pleon reflexed back), anterolateral margin of head with pair of notches in female (anterolateral margin of head without notches), pereopods 5 and 6 with large gap in female (without large gap), pleotelson with mushroom-shaped end in female (pleotelson with clavate end) and pleotelson subovate in male (posterior end of pleotelson obtusely pointed).

The present species is similar to *A. takanoshimensis* as reported by Markham (1982) being parasitic on *Diogenes edwardsii*. The present species can be distinguished by the following characters (those of *A. takanoshimensis*, as reported by Markham (1982), in parentheses): anterolateral margin of head with pair of notches in female (without notches), meri of pereopod 1 each with broad and long projection ventrally in female (without projection), pleotelson with mushroom-shaped end (round posterior end). *Athelges takanoshimensis* as reported by Markham (1982) also differs from the original description by Ishii (1914) in the following characters (those of original description of *A. takanoshimensis* in parentheses): pleon extended posteriorly in female (pleon reflexed back), pereopods 5 and 6 with large gap in female (without large gap).

Only two of the 349 individuals in the present study were parasitized, and the parasitized rate is thus 0.57%.

##### DNA barcode.

A 658-bp fragment of the mitochondrial COI gene, a 482-bp fragment of the mitochondrial 16S rRNA gene, and a 724-bp fragment of the nuclear 18S rRNA gene from the holotype and paratype females were determined. The sequence data, including the standard barcoding region for animal species, has been deposited as a DNA barcode of *A. pileus* sp. nov. in the GenBank under accession number LC876712.1, LC876713.1, LC876714.1, LC876715.1, LC876786.1, LC876787.1.

##### Etymology.

Derived from the Latin noun “pileus”, referring to the pleotelson, which is shaped like a mushroom cap.

The Japanese name comes from the pleotelson, which is shaped like a mushroom cap, Kinoko-o.

##### Phylogenetic analysis.

**Figure 7.**
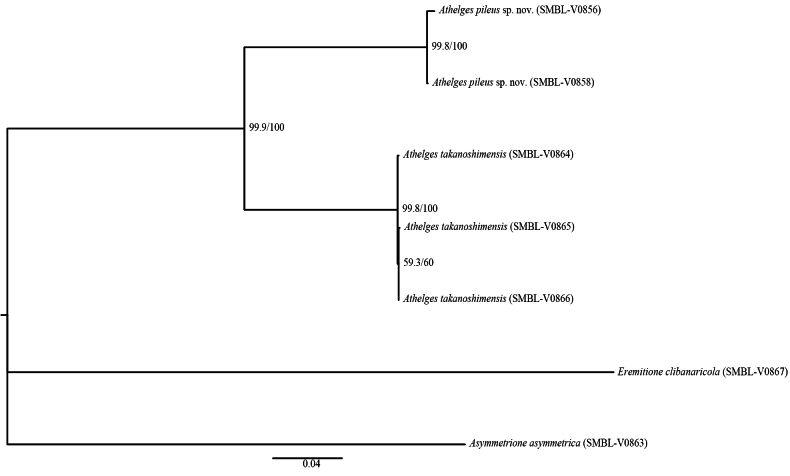
Maximum-likelihood tree from IQ-TREE analysis based on concatenated 16S, 18S, and COI sequences (1751 bp). Numbers near nodes indicate SH-aLRT and UFBoot support values. Clades with SH-aLRT ≥ 80% and UFBoot ≥ 95% are considered well supported.

ML tree, based on the concatenated dataset of 16S rRNA, 18S rRNA, and COI sequences (Fig. 7), supports the monophyly of *A. pileus* sp. nov. and *A. takanoshimensis* (SH-aLRT = 99.9% and UFBoot = 100%) and shows that these species are genetically distinct. These two sequences of *A. pileus* sp. nov. formed a fully supported clade (SH-aLRT = 99.8% and UFBoot = 100%), and three sequences of *A. takanoshimensis* also formed a clade with equally strong support (SH-aLRT = 99.8% and UFBoot = 100%). K2P distances among the two species were 20.7–21.1% (Table 3).

**Table 3.** K2P distances (%) among partial COI sequences (658 bp) from *A. pileus* sp. nov. and *A. takanoshimensis*.

	1	2	3	4	5
1. *A. pileus* sp. nov. (SMBL-V0856)					
2. *A. pileus* sp. nov. (SMBL-V0857)	0.8				
3. *A. takanoshimensis* (SMBL-V0864)	20.7	20.9			
4. *A. takanoshimensis* (SMBL-V0865)	20.9	21.1	0.5		
5. *A. takanoshimensis* (SMBL-V0866)	20.7	20.9	0.3	0.2	

## Discussion

Based on morphological observations, *Athelges pileus* sp. nov. can be recognized as a new species. Terminal shape of the pleotelson can be quite variable in *A. takanoshimensis* (e.g. Boyko 2004). However, terminal shape of the pleotelson of the present species did not match that of original descriptions of species belonging to *Athelges*, and therefore we recognized the present species as undescribed. Phylogenetic analyses further distinguished it from *A. takanoshimensis*, the only previously known Asian species of the genus. The specimen reported as *A. takanoshimensis* by Markham (1982) may represent an undescribed species, distinct from both *A. pileus* sp. nov. and *A. takanoshimensis*. It is suggested that *A. takanoshimensis* may possess parasitic adaptability and the potential for overlooked cryptic diversity. Future studies combining morphological and molecular data will be essential to clarify species boundaries within *Athelges*, especially considering the possibility that some previously reported records may represent undescribed species. Expanded geographic and host sampling across under sampled regions, particularly Asia, is also needed to assess the true diversity and biogeography of the genus *Athelges*.

### Key to species of *Athelges* based on females (Amended An et al. 2022)

**Table:** 

1	With biramous uropods	***A. prideauxii* Giard & Bonnier, 1890**
–	Without biramous uropods	**2**
2	Terminal part of pleotelson produced into uniramous bifurcated uropods	**3**
–	Terminal part of pleotelson entire	**6**
3	Pleomere 5 with tuberculate pleopod	***A. bilobus* Sars, 1898**
–	Pleomere 5 without pleopod	**4**
4	Terminal part of pleotelson with mushroom-shaped end	***Athelges pileus* sp. nov**.
–	Terminal part of pleotelson produced into uniramous bifurcated uropods	**5**
5	Pleotelson produced into two terminal semicircular uropods	***A. caudalis* Barnard, 1955**
–	Pleotelson produced into two reflexed anchor-shape uropods	***A. ankistron* Markham, 2010**
6	Pleotelson produced into a round posterior end	***A. takanoshimensis* Ishii, 1914**
–	Pleotelson produced into a pointed end	**7**
7	Exopodite and endopodite of pleopods with different size and shape	**8**
–	Exopodite and endopodite of pleopods with similar size and shape	**9**
8	Terminal part of pleotelson straight backward	***A. cladophorus* Gerstaecker, 1862**
–	Terminal part of pleotelson curved laterally	***A. tenuicaudis* Sars, 1898**
9	With sessile pleopods	**10**
–	With stem-like pleopods	**11**
10	Pleopods long and columniform	***A. aegyptius* Codreanu, Codreanu & Pike, 1965**
–	Pleopods short and foliate	***A. lacertosi* Pike, 1961**
11	Terminal part of pleotelson shorter than pleopods	***A. paguri* (Rathke, 1843)**
–	Terminal part of pleotelson much longer than pleopods	**12**
12	Body almost symmetrical	**13**
–	Body strongly asymmetrical	***A. pelagosae* Babić, 1912**
13	Body length shorter than 1 mm, pleopods elliptical	***A. guitarra* Giard & Bonnier, 1890**
–	Body length longer than 3 mm, pleopods foliate	***A. intermedia* (Anonymous, 1886)**

## Supplementary Material

XML Treatment for Athelges pileus

## Additional information

### Use of AI

No use of AI was reported.

### Author ORCIDs

Haruki Shinoda https://orcid.org/0009-0005-2086-5372

Hiroki Fujita https://orcid.org/0000-0003-4018-5727

Kenji Toyota https://orcid.org/0000-0001-8411-1389

Tomoyuki Nakano https://orcid.org/0009-0008-0109-1289

Michitaka Shimomura https://orcid.org/0000-0002-2524-7160

### Data availability

The type series is deposited in the Seto Marine Biological Laboratory (SMBL). The sequence data for *A. pileus* sp. nov. are available in GenBank.
